# Readmission of older patients after hospital discharge for hip fracture: a multilevel approach

**DOI:** 10.1590/S1518-8787.2016050005947

**Published:** 2016-04-15

**Authors:** Fátima de Lima Paula, Geraldo Marcelo da Cunha, Iúri da Costa Leite, Rejane Sobrino Pinheiro, Joaquim Gonçalves Valente

**Affiliations:** I Escola Nacional de Saúde Pública. Fundação Oswaldo Cruz. Rio de Janeiro, RJ, Brasil; II Instituto de Estudos em Saúde Coletiva. Universidade Federal do Rio de Janeiro. Rio de Janeiro, RJ, Brasil

**Keywords:** Aged, Hip Fractures, complications, Patient Readmission, Risk Factors, Quality of Health Care, Multilevel Analysis

## Abstract

**OBJECTIVE:**

To identify individual and hospital characteristics associated with the risk of readmission in older inpatients for proximal femoral fracture in the period of 90 days after discharge.

**METHODS:**

Deaths and readmissions were obtained by a linkage of databases of the Hospital Information System of the Unified Health System and the System of Information on Mortality of the city of Rio de Janeiro from 2008 to 2011. The population of 3,405 individuals aged 60 or older, with non-elective hospitalization for proximal femoral fracture was followed for 90 days after discharge. Cox multilevel model was used for discharge time until readmission, and the characteristics of the patients were used on the first level and the characteristics of the hospitals on the second level.

**RESULTS:**

The risk of readmission was higher for men (*hazard ratio* [HR] = 1.37; 95%CI 1.08–1.73), individuals more than 79 years old (HR = 1.45; 95%CI 1.06–1.98), patients who were hospitalized for more than two weeks (HR = 1.33; 95%CI 1.06-1.67), and for those who underwent arthroplasty when compared with the ones who underwent osteosynthesis (HR = 0.57; 95%CI 0.41–0.79). Besides, patients admitted to state hospitals had lower risk for readmission when compared with inpatients in municipal (HR = 1.71; 95%CI 1.09–2.68) and federal hospitals (HR = 1.81; 95%CI 1.00–3.27). The random effect of the hospitals in the adjusted model remained statistically significant (p < 0.05).

**CONCLUSIONS:**

Hospitals have complex structures that reflect in the quality of care. Thus, we propose that future studies may include these complexities and the severity of the patients in the analysis of the data, also considering the correlation between readmission and mortality to reduce biases.

## RESUMO

**OBJETIVO:**

Identificar as características individuais e hospitalares associadas ao risco de readmissão em idosos internados por fratura proximal do fêmur no período de 90 dias após a alta.

**MÉTODOS:**

Para a obtenção dos registros de óbitos e das readmissões foi realizado *linkage* dos bancos de dados do Sistema de Informações Hospitalares do Sistema Único de Saúde e do Sistema de Informações sobre Mortalidade do município do Rio de Janeiro dos anos de 2008 a 2011. A população de 3.405 indivíduos com 60 anos ou mais, com internação não eletiva por fratura proximal do fêmur, foi acompanhada por 90 dias após a alta. Foi utilizado o modelo de Cox multinível para o tempo da alta até a readmissão, sendo as características dos pacientes consideradas no primeiro nível e as características dos hospitais no segundo nível.

**RESULTADOS:**

O risco de readmissão foi maior para os homens (*hazard ratio* [HR] = 1,37; IC95% 1,08–1,73), indivíduos com mais de 79 anos (HR = 1,45; IC95% 1,06–1,98), pacientes que permaneceram internados por mais que duas semanas (HR = 1,33; IC95% 1,06-1,67) e para os que foram submetidos à artroplastia durante a internação quando comparados aos submetidos à osteossíntese (HR = 0,57; IC95% 0,41–0,79). Além disso, pacientes internados em hospitais estaduais tiveram menor risco para readmissão quando comparados aos internados nos hospitais municipais (HR = 1,71; IC95% 1,09–2,68) e federais (HR = 1,81; IC95% 1,00–3,27). O efeito aleatório dos hospitais no modelo ajustado manteve-se estatisticamente significativo (p < 0,05).

**CONCLUSÕES:**

Os hospitais apresentam estruturas complexas que refletem na qualidade da assistência. Assim, propõe-se que futuros estudos possam incluir essas complexidades e a gravidade dos pacientes nas análises dos dados, considerando também a correlação existente entre a readmissão e a mortalidade para reduzir vieses.

## INTRODUCTION

The population aging process has occurred in Brazil in a much more accelerated pace than the observed in developed countries. Due to morphological, biochemical, and psychological changes resulting from the aging process, older adults are more affected by chronic diseases, making hospital admissions most frequent. In addition, when inpatients, the older adults tend to lose their functional capacity more quickly and to remain more time hospitalized, becoming more susceptible to adverse events both during and after hospitalization^14^, which contributes to readmission and death.

The hip fracture (HF) is a cause of hospital admission that represents a huge risk for readmission, mainly among the older adults^1^. Although there is little information about the rates of readmission of older adults for HF in Brazil, in other countries these rates ranged from 18.3% in 30 days^6^ to 32.0% in six months^1^ and in one year^16^. In a three-month period were reported values from 16.1%^7^ to 19.0%^9^. Factors, such as sex, age, presence of comorbidities and length of stay in the first hospitalization, are associated with higher risk for readmission^6^ ^,^ ^7^ ^,^ ^16^. There is evidence that a significant proportion of readmissions could be avoided with improvements in the quality of both inpatient and outpatient care^8^. In a systematic review, it was observed a large hospital heterogeneity in the proportions of readmissions deemed avoidable ranging from 5.0% to 78.0%, with an average of 27.1%^18^.

A study performed with more than 6,000 community-dwelling older adults with HF in Paris^16^ showed that older people admitted to university hospitals presented a risk of readmission 14.0% lower than those admitted to public hospitals (p = 0.01). This difference is partially explained by the presence of a multidisciplinary team with participation of geriatricians in university hospitals. We can observe, then, that differences in conduct due to the quality of care are associated with hospital readmissions.

Thus, since differences in treatment may influence the risk of readmission, variables of hospitals should be included in the analyses on re-hospitalization of patients being discharged after undergoing procedures for HF. In this context, whereas the degree of heterogeneity among the hospitals cannot be explained by the variables available, it is necessary to consider a model that includes random effects for the hospitals.

The present study aimed to identify individual and hospital features associated with the risk of readmission in older inpatients for hip fracture (HF) within 90 days after discharge.

## METHODS

This is a retrospective longitudinal study, with passive follow-up of older adults aged 60 or older, discharged after hospitalization for correction of HF, in hospitals in the Unified Health System (SUS) of the city of Rio de Janeiro, between 2008 and 2011. The information regarding admissions and readmissions was obtained based in the information of *Autorização de Internação Hospitalar* (AIH – Hospitalization Authorization) of the Hospital Information System of SUS (SIH-SUS) in the city of Rio de Janeiro, provided by the Municipal Health Department of Rio de Janeiro. Data related to deaths that occurred among the older adults after discharge, used for the calculation of the period of exposure to risk of readmission, were obtained from the Mortality Information System (SIM) between 2008 and 2011.

During this period, 231,056 hospitalizations were observed in the database of the AIH, and 163,842 deaths at the SIM database. Originally, all 3,582 patients of 60 years old or older met eligibility criteria, which were to reside in the municipality of Rio de Janeiro, with non-elective hospitalization due to HF (S720, S721, S722, according to the International Classification of Diseases and related health problems, 10^th^ revision – ICD-10), in hospitals of the SUS in the city, with discharge between January 2008 and December 2011, and who remained at least for 24h in the hospital. A hundred and twenty-eight (3,6%) were excluded from the cohort, of which 75 were transferred to another hospital; 26 were admitted in hospitals with less than 20 hospitalizations, and 27 were cases of polytrauma or hospitalization due to multiple surgeries. This process resulted in a population of 3,454 older adults with admission due to HF.

Readmissions and deaths observed after discharge were then identified by linkage^10^ based on the probabilistic relationship, since the databases from SIH-SUS and SIM do not have a patient’s common identifier. To reduce the linkage errors and optimize the pairing of the records, the databases were cleaned and standardized. The next step was blocking^2^, a step in which blocks are used by means of field combinations (name, year of birth, sex) in such a way that the comparison between the databases can be performed. The variables address and mother’s name were used to help at the moment of manual review. At this point, we identified 49 cases of hospitalization that showed inconsistency in the recording of dates, and the date of hospitalization was overlaid to the date of another hospitalization. The final population was composed of 3,405 older adults after discharge from HF hospital admission. The outcome of interest was the elapsed time between the discharge for correction of the HF and the first hospitalization that occurred during the period of 90 days. This period was elected because 80.0% of the readmissions that occurred within 90 days would be in fact associated with the first hospitalization^16^. Older adults who were not readmitted or who died in the period of 90 days were censored.

The individual variables of patients considered in the analysis were: age (60-69 years, 70-79 years, and ≥ 80 years); type of fracture (S720 – femoral neck fracture, S721 – pertrocantheric fracture, S722 – subtrochanteric fracture); length of stay of first hospitalization in weeks (≤ 2, > 2), and type of surgery (partial and total arthroplasty, cemented and uncemented; osteosynthesis – surgical treatments excluding arthroplasties; conservative treatments – non surgical). The variables of the hospitals obtained from *Cadastro Nacional de Estabelecimentos de Saúde* (CNES – National Register of Health Establishments) were: the administrative level (municipal, state, and federal), number of beds, emergency service, outpatient traumatology and orthopedics service, and outpatient physiotherapy service.

Since hospital care received in different hospitals can exert different effects on the readmission of a patient, but information about that care is unavailable in the SIH-SUS database, a multilevel approach was used in the analysis of the data. This approach assumes that there is a correlation between the times for readmission of patients who were discharged from the same hospital. The Cox multilevel proportional hazards model^3^ was used, assuming the patients in the first level and the hospitals in the second level. The risk of readmission in time *t* of the patient *i* that was discharged from a hospital *j* can be expressed by:


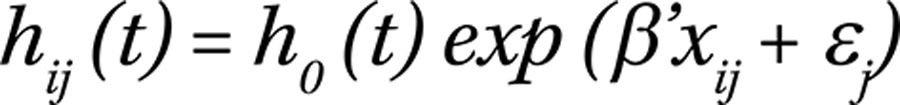


where: *x*_*ij*_ is a set of independent variables*; h*_o_ *(t)* is the baseline hazard function, and ε_*j*_ is the random effect of the hospital to which it is assumed to have a normal distribution, with zero mean and variance σ^2^.

The analysis of the Cox multilevel proportional hazards model was developed in two stages: in the first one, the effect of each variable was separately observed in the presence of random effects (crude or unadjusted model), having selected all the variables that presented p < 0.20 in multivariate analysis. At this stage, only the significant variables at the level of 5% (p < 0.05) were included in the final model. The proportional hazards assumption was evaluated by checking the Schoenfeld residuals^15^. The functional form of the model and the outlier points were analyzed by Martingale residuals^17^. The confidence intervals of the risks associated with hospitals were estimated by the method Bootstrap^4^ ^,^ ^5^.

All statistical analyses were performed using the software R (The R Project for Statistical Computing)^a^, with the data linkage being performed with the aid of the package *RecordLinkage* ^b^ and the survival analysis, with packages *Survival* ^c^ and *Coxme* ^d^.

This study was approved by the Research Ethics Committee of the Escola Nacional de Saúde Pública (CAAE-07040412.0.0000.5240 – Ordinance 119.827) and by the Research Ethics Committee of the Municipal Health Secretariat of Rio de Janeiro (Protocol 77/12, Ordinance 280A/2012).

## RESULTS

Of the 3,405 older adults participating in the study, more than 2/3 were women (71.9%) and almost half were 80 years old or more (48.2%). Osteosynthesis was used for most of the cases (64.3%) and the most common type of fracture was the femoral neck (55.4%). Municipal level hospitals had the higher number of admissions (60.0%). More than half of the hospitals had outpatient traumatology and orthopedics service, and about 82.0% had outpatient physiotherapy service (Table 1).

**Table 1:** Characteristics of the population of older adults who were discharged after hospitalization for hip fracture. Rio de Janeiro, RJ, Southeastern Brazil, 2008-2011. (N = 3,405)

Characteristic	n	%
Sex		
Female	2,447	71.9
Male	958	28.1
Age (years old)		
60-69	593	17.4
70-79	1,171	34.4
80 or more	1,641	48.2
Procedure		
Osteosynthesis	2,189	64.3
Arthroplasty	1,032	30.3
Conservative	184	5.4
Fracture type		
Femur neck (S720)	1,885	55.4
Pertrocantheric (S721)	1,196	35.1
Subtrocantheric (S721)	324	9.5
Permanence time		
≤ 2 weeks	1,556	45.7
> 2 weeks	1,849	54.3
Hospital administrative level		
Municipal	2,043	60.0
State	1,030	30.2
Federal	332	9.8
Number of beds		
140-250	560	16.4
250-300	1,645	48.3
300-411	1,200	35.3
Admission in hospital with outpatient traumatology service		
Yes	1,866	54.8
No	1,539	45.2
Admission in hospital with outpatient physiotherapy service		
Yes	2,787	81.9
No	618	18.1

Overall, 333 HF patients (9.8%) were readmitted within 90 days, being 15.9% female and 17.8% male. The mean age was 78.4 (SD = 8.7). The mean length of stay after discharge for hip fracture hospitalization was 19.0 days (SD = 13.9) and they were respectively 21.8, 16.9, and 24.0 days in state, municipal, and federal hospitals. During the 90-day follow-up, 227 deaths occurred (6.7%). State hospitals had the highest percentage of deaths (8.0%), and federal hospitals had the lowest percentage (4.6%). All hospitals had emergency service.

Table 2 presents the variables that were statistically significant in the level of 20.0%. The risk of readmission was higher among men, patients over the age of 79 years old, and patients who remained hospitalized for more than two weeks. Patients undergoing internal fixation showed lower readmission risk compared with those who underwent arthroplasty. Federal and municipal hospitals presented readmission risk about twice greater than the one observed in state hospitals. The assumption of proportional risks has been verified for all variables included in the final model. Figure (A) presents the risk of readmission and respective confidence intervals of 95% based on the results of the adjusted model including only the random effects from hospitals. State hospitals presented the lowest risks, all of which are statistically significant. Figure (B) shows the random effects of hospitals controlling by the variables included in the final model: gender, age, administrative type of hospital, length of stay, hospital procedure, and random effect of hospitals. We identified a reduction in the effects of the hospitals and their confidence intervals of 95% when controlled by the other variables. Although the confidence intervals have the value of 1 after adjusting by the set of variables, the random effect of the hospitals in the adjusted model remained statistically significant (p < 0.05).

**Table 2:** Crude and adjusted readmission hazards, considering random effect. Older adults discharged after hospitalization due to hip fracture. Rio de Janeiro, RJ, Southeastern Brazil, 2008-2011.

Characteristic	Gross HR	95%CI	Adjusted HR*	95%CI
Sex				
Female	1.00		1.00	
Male	1.28	1.02–1.61	1.37	1.08–1.73
Age (years old)				
60-69	1.00		1.00	
70-79	0.98	0.70–1.37	1.04	0.74–1.45
80 or more	1.31	0.96–1.78	1.45	1.06–1.98
Permanence time				
Up to two weeks	1.00		1.00	
More than two weeks	1.31	1.04–1.65	1.33	1.06–1.67
Procedure				
Arthroplasty	1.00		1.00	
Osteosynthesis	0.61	0.49–0.77	0.57	0.41–0.79
Conservative	0.93	0.58–1.49	0.91	0.56–1.49
Fracture type				
Femur neck	1.00		1.00	
Pertrocantheric	0.73	0.57–0.94	1.06	0.75–1.49
Subtrocantheric	0.83	0.57–1.21	1.19	0.76–1.87
Hospital Administrative Scope				
State	1.00		1.00	
Municipal	2.00	1.36–2.95	1.71	1.09–2.68
Federal	2.26	1.38–3.72	1.81	1.00–3.27
Attended in hospital with outpatient traumatology service				
No	1.00		1.00	
Yes	1.74	1.11–2.74	1.19	0.77–1.84

HR: hazard ratio

* Adjusted according to sex, age, length of stay, procedure, and random effect of hospitals.

## DISCUSSION

Among the older adults, hospitalization for hip fracture was more incident in women than in men, but the risk of readmissions was higher among men. State hospitals presented less risk of readmission and higher risk of death.

Approximately 10.0% of the patients were readmitted within 90 days after discharge, a percentage smaller than the one found in studies on other countries^7^ ^,^ ^9^, which are around 18.0%. A hypothesis for this finding is that part of readmissions occurs in private hospitals or in hospitals in other cities, which are not included in the SIH database. Another hypothesis would be the loss of readmissions in the process of linkage, since the efficacy of the method depends on the quality of the data. In addition, lower readmission rate could be explained by difficult access to the health service, causing older adults’ death, for the delay in attendance, before they were readmitted. Studies on readmission of older adults due to HF do not analyze deaths after discharge, which impairs the confirmation of this hypothesis. Thus, we must be cautious when interpreting these results, for, in order to assess the quality of hospital care, the interdependence between mortality and readmission cannot be ignored^11^.

According to the literature, a higher risk of readmission among male patients with older age was identified^6^ ^,^ ^7^ ^,^ ^16^. The Diretrizes de Saúde da Pessoa Idosa (Older Adults Health Guidelines)^e^ consider frail older people those who are 80 years or older, and cite as clinical signs of fragility sarcopenia, osteopenia and changes of gait and balance, among other problems, which make them vulnerable to negative outcomes. In other words, older adults aged 80 years or older present more musculoskeletal and balance impairments that hinder rehabilitation, and thus predispose to new fractures, thereby increasing the risk of readmissions.

The length of stay has been used as an indicator of hospital efficiency and a replacement cost measure related to quality of care, although this relationship is not well established^11^. The higher readmission risk among patients with a greater length of stay can be justified by the severity of the patient status or by the poor quality of care^f^. In Canada, a measure used to assess the quality of the access to the health service is the waiting time until the surgery of the older adults admitted for HF. Although it is admitted that the waiting time to surgery can be influenced by comorbidities or different practices of hospitals, the increase in that time is also attributed to the lack of resources and the lack of professionals, or other aspects related to access to care^g^.

Arthroplasty presented a higher risk of readmission when compared with osteosynthesis. The surgical procedure depends on the type of fracture^h^. To femoral neck fracture, for instance, Nicolaides et al.^12^ found that arthroplasty had the lowest percentage of readmission to a second surgery than osteosynthesis. In another study, Rogmark et al.^13^ found that readmissions in 180 days related to complications of hip joint were more frequent in patients receiving internal fixation than in those who underwent arthroplasty.

It is expected that the features and quality of hospital care are associated with readmission in such a way that patients in the same hospital have a similar profile, which makes these data correlated. In the Cox multilevel proportional risk model, considering the structure of dependency among patients admitted to the same hospital, the inclusion of random effects enabled to assess the risks associated with each of the hospital units. It was observed that two hospitals, one from the federal level and the other from the municipal level, presented a greater risk than the others, even after adjusting by a set of variables.

Much of the variability of the hospitals was explained by the hospital administrative type. This scope may be capturing differences related to problems of access to health services, the poor quality of care provided, or even to the precariousness of the hospital structure that affect the risk for readmission. State hospitals presented less risk of re-hospitalization in relation to municipal and federal ones, but also presented a higher proportion of deaths, indicating that lower readmission rate among state hospitals does not necessarily imply a better quality of the care provided.

No information about hospitals that could justify the association between the administrative type and the risk of readmission among the older adults was found, as occurred in the study of Teixeira et al.^16^, in which the multidisciplinary team with geriatrics played an important role in reducing the risk of readmission. In Brazil, the State Networks of Healthcare for the Older Adults^i^ consider necessary the presence of multidisciplinary and interdisciplinary teams in the care of older adults. The deployment of these networks, providing differentiated service for older people in relation to physical facilities, equipment, and human resources, did not seem to be effective in practice.

There are two possibilities for the antagonistic association between readmissions and the presence of outpatient traumatology and orthopedics service. This service can allow monitoring of the patient after discharge, reducing the rate of readmissions of older people admitted in these hospitals. On the other hand, it can better identify the problems arising from hospitalization and increase the number of readmissions. The crude hazard pointed to the second hypothesis, but this variable was not statistically significant in the adjusted model.

This study has some limitations. First, data did not inform features due to hospital care. Second, we do not have information of the participants’ comorbidities neither the time gap between the fracture and inpatient care. Another limitation is that private hospitals and hospitals in other counties were not used to search for the readmissions.

The health system in Brazil differs from the system of other countries. As far as we are concerned, this is the first study on readmissions for patients after hospitalization for hip fracture performed with Brazilian older people. From the association between the hospital type and readmission, and the statistical significance of the random effect of hospitals, we noted the importance of studies that might explain hospital differences. Thus, we found evidence of the necessity of variables with information about comorbidities in the SIH-SUS database and more detailed information about hospitals in the National Register of Health Establishments (CNES).

**Figure:**
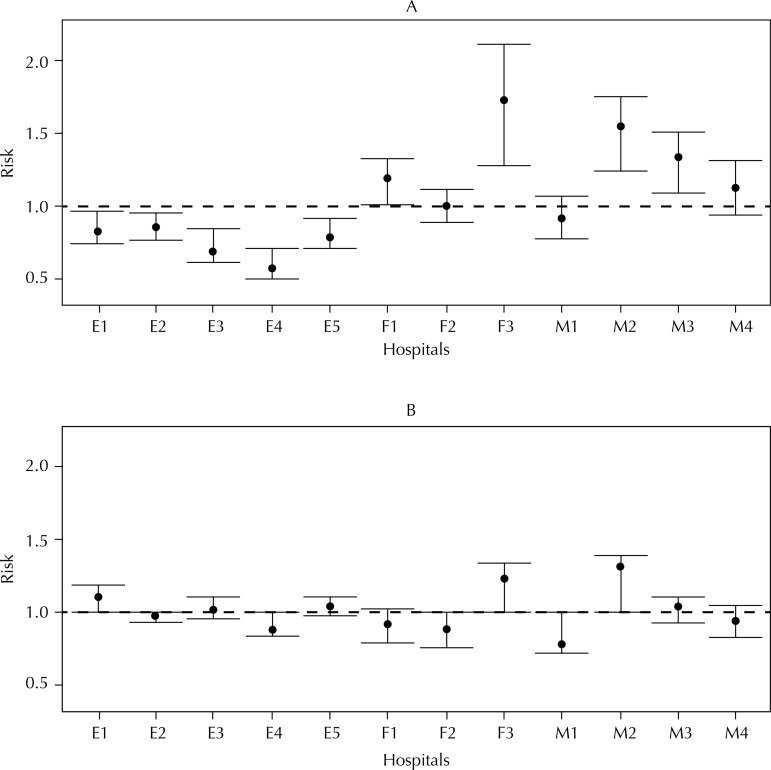
Risk of readmission and confidence interval of 95% for each hospital. A. Model with random effects of hospitals only. B. Model adjusted for sex, age, procedure, scope, length of stay, and the random effects of hospitals. E: State scope; M: Municipal scope; F: Federal scope

## Footnotes

a R Core Team. R: a language and environment for statistical computing [software]. Vienna; R Foundation for Statistical Computing; 2013 [cited 2015 Oct 10]. Available from: http://www.R-project.org/

b Borg A, Sariyar M. Package RecordLinkage. Mainz; 2012. Available from: http://cran.r-project.org/web/packages/RecordLinkage/index.html

c Therneau TM. A Package for survival analysis in S. R package; version 2.37-7; 2014. Available from: http://cran.r-project.org/web/packages/survival/index.html

d Therneau TM. Mixed effects Cox models; version 2.2-3; 2014. Available from: http://cran.r-project.org/web/packages/coxme/index.html

e Secretaria de Estado da Saúde (ES). Diretrizes de Saúde da Pessoa Idosa. Vitória, ES: Secretaria de Estado da Saúde; 2008 [cited 2013 Dec 12]. Available from: http://docslide.com.br/download/link/diretrizes-de-saude-da-pessoa-idosa-espirito-santo-2008

f Nolte E, Roland M, Guthrie S, Brereton L. Preventing emergency readmissions to hospital: a scoping review. Santa Monica: RAND Corporation; 2012 [cited 2013 Nov 10]. Available from: http://www.rand.org/content/dam/rand/pubs/technical_reports/2012/RAND_TR1198.pdf

g Canada. Statistics Canada: health system performance. Ottawa; 2013 [cited 2014 Apr 13]. Available from: http://www.statcan.gc.ca/pub/82-221-x/2013001/def/def3-eng.htm

h Butler M, Forte M, Kane RL, Joglekar S, Durval SJ, Swiontkowski M et al. Treatment of common hip fractures. Rockville: Agency for Healthcare Research and Quality (US); 2009 [cited 2015 Oct 10]. Introduction. Available from: http://www.ncbi.nlm.nih.gov/books/NBK32595

i Ministério da Saúde. Portaria MS nº 702 de 12 de abril de 2002. Dispõe sobre a criação de mecanismos para a organização e implantação de Redes Estaduais de Assistência à Saúde do Idoso. Brasília (DF); 2002 [cited 2015 Oct 10]. Available from: http://www.camara.gov.br/sileg/integras/239984.pdf
